# Predicting drought tolerance from slope aspect preference in restored plant communities

**DOI:** 10.1002/ece3.2881

**Published:** 2017-03-30

**Authors:** Sarah Kimball, Megan E. Lulow, Kathleen R. Balazs, Travis E. Huxman

**Affiliations:** ^1^ Center for Environmental BiologyUniversity of CaliforniaIrvineCA 92697‐1450USA; ^2^ Irvine Ranch ConservancyIrvineCAUSA; ^3^ Center for Environmental Biology and Department of Ecology and Evolutionary BiologyUniversity of California, IrvineIrvineCAUSA

**Keywords:** coastal sage scrub, drought response, grassland, microhabitat preference, restoration

## Abstract

Plants employ strategies of tolerance, endurance, and avoidance to cope with aridity in space and time, yet understanding the differential importance of such strategies in determining patterns of abundance across a heterogeneous landscape is a challenge. Are the species abundant in drier microhabitats also better able to survive drought? Are there relationships among occupied sites and temporal dynamics that derive from physiological capacities to cope with stress or dormancy during unfavorable periods? We used a restoration project conducted on two slope aspects in a subwatershed to test whether species that were more abundant on more water‐limited S‐facing slopes were also better able to survive an extreme drought. The attempt to place many species uniformly on different slope aspects provided an excellent opportunity to test questions of growth strategy, niche preference, and temporal dynamics. Perennial species that established and grew best on S‐facing slopes also had greater increases in cover during years of drought, presumably by employing drought tolerance and endurance techniques. The opposite pattern emerged for annual species that employed drought‐escape strategies, such that annuals that occupied S‐facing slopes were less abundant during the drought than those that were more abundant on N‐facing slopes. Our results clarify how different functional strategies interact with spatial and temporal heterogeneity to influence population and community dynamics and demonstrate how large restoration projects provide opportunities to test fundamental ecological questions.

## 1 Introduction

Droughts in semi‐arid regions are becoming increasingly common and severe in recent decades as compared to historical climate records (AghaKouchak, Cheng, Mazdiyasni, & Farahmand, 2014; Dai, 2013). Plant ecologists have documented wide‐spread drought‐induced mortality in some species and are motivated to predict how plant communities and their component species may respond to future increases in drought frequency and severity (Allen et al., 2010; Hufnagel & Garamvolgyi, 2014; McAuliffe & Hamerlynck, 2010; Sperry & Love, 2015). One possibility is the species that occupy more arid microclimates may be able to withstand drought better than species that occupy mesic microclimates (Casper, 1996; Ramirez, Rada, & Llambi, 2015). Given projections of future climate where existing water‐limited regions may experience more negative water balance (Weltzin et al., 2003), this possibility would predict vegetation transitions to more xeric species over time. Understanding such vegetation change is important to making assessments of integrated Earth System response to climate and the future of biodiversity.

Plants exhibit different strategies for dealing with arid conditions, historically described as drought tolerance, endurance, escape, and avoidance, with some disagreement in the literature as to exact groupings (Gutschick, 1987; Shantz, 1927; Smith, Monson, & Anderson, 1997). Drought‐tolerant plants typically have traits such as high water‐use efficiency or the ability to adjust cell water relations to maintain critical turgor pressure enabling survival through xeric periods (Jacobsen, Pratt, Davis, & Tobin, 2014; Jones, 1992; Pratt et al., 2012, 2014). Drought‐endurance strategies include drought‐deciduous plants that reduce evaporative surfaces during drought, ceasing to photosynthesize, yet maintaining additional perennial structures (Havstad & Schlesinger, 2006; Kozlowski, 1976; Shantz, 1927). Drought‐avoidance strategies include traits such as deep roots that enable species to continue to access water through dry periods (Havstad & Schlesinger, 2006; Kozlowski, 1976), although this strategy is sometimes categorized as drought‐tolerance (Franks, 2011). Annual plants are considered drought‐escapers because they have traits that enable them to escape dry periods such as early season flowering or prolonged seed dormancy (Jones, 1992). Jones (1992) points out that plants may exhibit traits that put them in more than one category, and suggests researchers shift to focusing on mechanisms, recognizing that species may employ more than one strategy for coping with drought. Indeed, species may utilize tolerance‐categorized traits in response to water limitation occurring over short time‐scales, but traits associated with escape in response to variance in drought over extended periods (Kimball, Gremer, Angert, Huxman, & Venable, 2012). Conventional wisdom holds that plants with traits that enable them to tolerate dry microclimates may also be able to withstand dry periods in time—in fact, many modern, niche‐based species distribution models assume such space‐time predictions to be robust (Scheiter, Langan, & Higgins, 2013).

One major landscape feature influencing microclimate and potential niche opportunity for vegetation is slope aspect. Slope aspect influences incident solar radiation, which in turn alters soil and air temperature, changing the driving force for water flux to the atmosphere and affecting soil moisture (Fekedulegn, Hicks, & Colbert, 2003). Plant communities in semi‐arid regions frequently differ in composition on slopes with different aspects, such that the polar‐facing slope aspects are characterized by more lush, evergreen vegetation than equatorial‐facing slopes, which contain more open, drought‐deciduous, shrubby vegetation (Ackerly, Knight, Weiss, Barton, & Starmer, 2002; Armesto & Martinez, 1978). As plants influence processes such as runoff, soil moisture, and erosion, there may also be plant‐environment feedbacks that lead to more extreme abiotic differences between slopes with different aspects (McAuliffe et al., 2014). Slope aspect influences fire regimes, with more frequent fires on drier slopes (Beaty & Taylor, 2001). Overall, plants that prefer more xeric slope aspects are characterized by traits that enable them to survive the reduction in growth potential and increase in physiological stress brought on by lower soil moisture, which would likely also enable them to survive droughts (Ackerly et al., 2002; Hamerlynck & Huxman, 2009; Nobel & Linton, 1997).

Large‐scale restoration projects in which species are seeded or planted at large scales for mitigation and conservation across varying microclimates are being conducted worldwide (Gerla, Cornett, Ekstein, & Ahlering, 2012; Hagen & Evju, 2013; Lengyel et al., 2012; Thompson, 2011; Trabucchi, Ntshotsho, O'Farrell, & Comin, 2012). These landscape‐scale manipulations provide opportunities to study strategies for establishment and growth under varying environmental conditions (Howe & Martinez‐Garza, 2014), and can be used to replicate plant communities in space, providing information regarding microhabitat preferences (Howe & Martinez‐Garza, 2014). The influence of environmental variation in space and time is not often considered in the contractual framework guiding restoration projects, yet both slope aspect and drought have strong influences on overall project success and the impact of limited funds to support biodiversity protection (Alday, Marrs, & Martinez‐Ruiz, 2010; Kimball et al., 2015). Here, we use data from an extensive 25‐ha, experimental restoration project in which N‐ and S‐facing slopes were similarly seeded with a diverse set of native species from two co‐occurring plant communities to determine whether preference for S‐facing slope aspects predicted performance in time. The implementation and monitoring of this project coincided with the largest drought in the region's contemporary record (Shukla, Safeeq, AghaKouchak, Guan, & Funk, 2015). We asked how the % cover of species in the West Loma Restoration Experiment varied depending on the slope aspect, and how species changed in cover across three years of severe drought. Was the response to slope aspect related to the response to drought? We hypothesized that the species that preferred S‐facing slopes would have less severe responses to drought conditions through time and vice versa. Our results will help to clarify the relationship between spatial and temporal niche dynamics, focusing on major differences in plant life histories that can then be used to understand how different combinations of traits determine plant performance in varying environments. We also provide an example of how restoration projects may be used in experimental contexts to further understanding of fundamental ecological concepts.

## 2 Materials and Methods

### 2.1 Restoration experiment

The restoration project, the West Loma Ecological Restoration Experiment (Kimball, Funk, Sandquist, & Ehleringer, 2016 *;* Kimball et al., 2015), was conducted in a 25‐ha subwatershed in the Santa Ana Mountains of Orange County, California, USA. The climate is Mediterranean, and native vegetation in the surrounding area consists of a prairie‐shrubland (California grassland – coastal sage scrub) mosaic. Our study site was initially dominated by exotic Eurasian grass species, which we reduced in abundance by a combination of several site preparation techniques initiated in 2009, including the application of herbicide, mowing, and manual removal. This site preparation was followed by seeding of natives in plots with seven different combinations of native species, or seed mixes, in 2011. The same seed mixes (Table 1) were replicated extensively on both the North‐ and the South‐facing slopes (*n *>* *100 for all slope‐seed mix combinations) in the large drainage in a complete block design (Appendix S1). Seeds of all species were added to plots across both slope aspects using the same methods (identical seed sowing techniques, seeding rate, time of seeding, maintenance techniques, etc.), so we consider differences in germination, establishment, and growth of each species between the two slope aspects to represent preferences for one slope aspect over the other. Although we recognize that microclimatic differences, competition, and other unknown factors likely also influenced establishment and growth, we consider significant differences in establishment depending on slope aspect to indicate aspect preference. The seeding rate used in this project is similar to that used in previous restoration projects in the area (Kimball, Lulow, Mooney, & Sorenson, 2014). Nonnative species were removed at regular intervals (every 4 weeks on average during the growing season) to reduce competition and increase the growth of natives. Despite this regular maintenance, there were several nonnative species that continued to establish in our study plots (Table 1). Plant establishment and growth depended on rainfall because there was no irrigation in the study area. Full details regarding the restoration, including a diagram of the block design with replicate seed mixes applied across the site, are provided in another publication (Kimball et al., 2015).

**Table 1:** (A) List of native species seeded in each community, by seed mix. Also listed is the life cycle (annual, perennial, or biennial) for each species, as well as the seeding rate, in PLS (pure live seeds) per pounds per hectare. Note that one native shrub, *Artemisia californica*, was included in the grassland forb mix at low levels, because it naturally occurs in transitional habitats at extremely low frequencies. (B) Nonnative species, the functional group to which they belong (grass or forb), and their life cycle (all annuals)

(A)				
Community	Seed mix	Species	Life cycle	PLS lbs/ha
Coastal Sage Scrub	Forb	*Deinandra fasciculata*	Biennial	1.9
		*Eschscholzia californica*	Biennial	4.9
		*Eucrypta chrysanthemifolia*	Annual	0.6
		*Malacothrix saxitilis*	Biennial	0.2
		*Phacelia cicutaria*	Annual	2.5
		*Salvia columbariae*	Annual	1.2
	Grass	*Elymus condensatus*	Perennial	7.4
		*Stipa pulchra*	Perennial	7.4
	Shrub	*Artemisia californica*	Perennial	2.5
		*Encelia californica*	Perennial	7.4
		*Eriogonum fasciculatum*	Perennial	7.4
		*Acmispon glaber*	Perennial	7.4
		*Mirabilis californica*	Short‐lived perennial	2.5
		*Salvia apiana*	Perennial	3.7
Grassland	Forb	*Artemisia californica*	Perennial	0.6
		*Castilleja exserta*	Annual	0.6
		*Cirsium occidental*	Short‐lived perennial	3.7
		*Deinandra fasciculata*	Biennial	1.9
		*Grindelia camporum*	Annual	7.4
		*Isocoma menziesii*	Perennial	1.2
		*Acmispon strigosus*	Annual	4.9
		*Lupinus microcarpus*	Annual	3.7
		*Lupinus succulentus*	Annual	6.2
		*Plantago erecta*	Annual	2.5
	Grass	*Stipa pulchra*	Perennial	29.7

(B)				
Nonnative species found in all plots	Functional group	Species	Life cycle	
	Forb	*Brassica nigra*	Annual	
	Grass	*Brachipodium distachyon*	Annual	
	Grass	*Bromus madritensis*	Annual	
	Forb	*Erodium cicutarium*	Annual	
	Forb	*Melilotus indicus*	Annual	
	Forb	*Salsola tragus*	Annual	
	Forb	*Sonchus oleraceus*	Annual	

### 2.2 Cover on N‐ and S‐facing slopes through time

Each May (late Spring at our study site), from 2012 to 2015, we used point‐intercept, with 24 points per 5 × 5 m plot, to determine the percent cover of all species. The percent cover of each species (arcsine‐transformed) was analyzed by mixed‐model, repeated measures ANOVA, with year, aspect, and the year‐by‐aspect interaction as fixed factors, and block as a random factor in each model. We used Tukey post hoc tests to investigate significant differences among specific treatment combinations. Previous analyses have indicated that each functional group (grass, forb, shrub) established best when seeded in separate strips rather than all functional groups mixed together (Kimball, Lulow, et al., 2014; Kimball et al., 2015). We used data from the seed mix with the highest cover of each native focal species (ex: shrub‐only or grass‐only) to analyze influence of aspect and cover through time. For the nonnatives, we used data from all plots.

### 2.3 Relationship between response to slope aspect and drought

Mean annual precipitation in this area is 30 cm, which primarily falls between October and April (Irvine Ranch weather 1902–2003; Western Regional Climate Center). Precipitation during the initial growing season (October 2010–April 2011) in which seeding occurred was 51 cm, and precipitation dropped to 17 cm (2011–12), 13 cm (2012–13), 12 cm (2013–14), and 17 cm (2014–15) in each of the following growing seasons, representing a severe drought (Irvine Ranch Weather Station).

For each species, we calculated log response ratios to drought (RR_drought_) and to slope aspect (RR_aspect_). May 2012 was the first season post‐seeding and the first season of cover data reflecting germination and initial establishment. By May 2015 the area had experienced four consecutive growing seasons of drought, so RR_drought_ was calculated as ln(cover in 2015/cover in 2012). Cover from both slope aspects were averaged for calculating RR_drought_. We calculated response to slope aspect (RR_aspect_) as ln(cover on S‐facing slopes/cover on N‐facing slopes). We used data from 2013 for calculation of RR_aspect_ because this allowed for one full year of growth, post‐seeding, sufficient time for all species to reproduce. The use of 2013 data allowed for plants to respond to abiotic differences between N‐ and S‐facing slopes, and data from other years resulted in similar RR_aspect_ values (Appendix S2). Linear regression analysis was used to determine whether RR_drought_ could be predicted from RR_aspect._ Plants with different life cycles (annuals, biennials, and short‐lived perennials with lifespans ≤3 years, hereafter simply referred to as “annuals and biennials” compared to perennials with longer lifespans) were analyzed separately after the relationship between RRs was observed to differ between these two groups.

We also calculated RRs for non‐native species that were regularly found in the study plots, either having established from seeds that dispersed into the restoration project from the surrounding landscape or from the seed bank. Although the non‐native species were being actively removed, the amount of time spent removing weeds from each plot was standardized across both slope aspects, so we expect that their cover values accurately reflect aspect preference. However, it is likely that the RR_drought_ values of non‐natives were influenced by control efforts, so we included provenance (native or non‐native) as a covariate in our analysis for annuals and biennials.

## 3 Results

### 3.1 Cover on N‐ and S‐facing slopes through time

**Figure 1:**
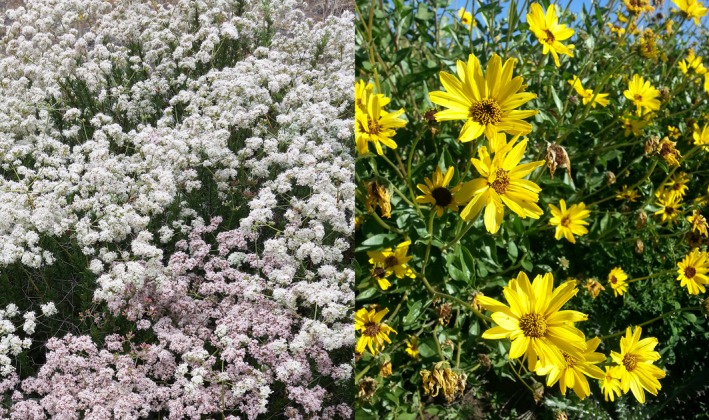
Photographs of *Eriogonum fasciculatum* (left) and *Encelia californica* (right), two of the shrub species seeded at the West Loma Ecological Restoration Experiment. *Eriogonum* was more abundant on S‐facing slopes and increased in cover during the drought, while *Encelia* was more abundant on N‐facing slopes and its cover increased very little during drought years

**Figure 2:**
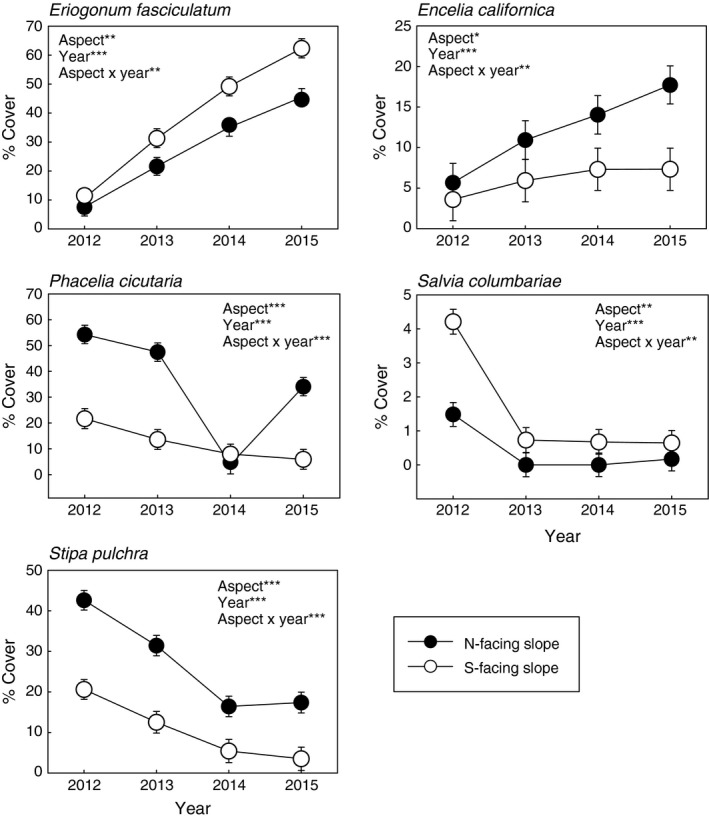
Average % cover of several abundant species through time on N‐facing and S‐facing slopes. Bars represent ± 1*SE*. We performed a repeated measure, mixed‐model ANOVA to determine whether % cover varied depending on year, slope aspect, or the interaction between the two factors. Significant factors are inserted in each graph, where *indicates *p *<* *.05, **indicates *p *<* *.01, and ***indicates *p *<* *.0001. The species with the highest overall cover for each functional group are presented here, and graphs of all other species can be found in Appendix S4. Results from ANOVAs of all species are provided in Appendix S3

The cover of all species changed significantly through time (Appendix S3). The majority of species (55%) had significantly higher cover on one slope aspect than another. For example, the shrub *Eriogonum fasciculatum* and the forb *Salvia columbariae* had higher cover on S‐facing slopes than N‐facing slopes, while the shrub *Encelia californica*, the forb *Phacelia cicutaria*, and the perennial bunchgrass *Stipa pulchra* had higher cover on N‐facing slopes (Figures 1 and 2). Significant slope aspect‐by‐year interactions indicated that the preference for one aspect over another was more pronounced in some years (those with higher cover) than in other years. Native species included in the seed mixes in Table 1 that had very low establishment (<1% average cover for every slope aspect‐year combination) were excluded from the analyses.

### 3.2 Relationship between response to slope aspect and drought

**Figure 3:**
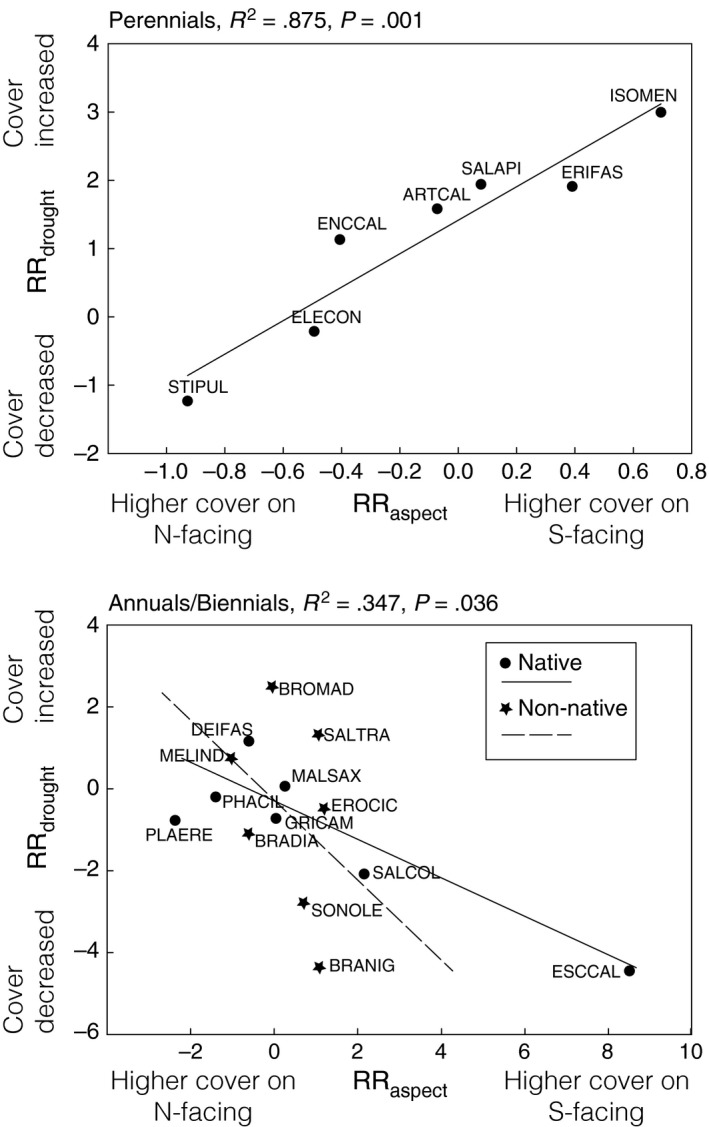
The relationship between RR_drought_ and RR_aspect_ for perennials (top panel) and for annuals and biennials (bottom panel). For each species, RR_drought_ = ln(cover in 2015/cover in 2012). RR_aspect_ = ln(cover on S‐facing slopes/cover on N‐facing slopes). We used data from 2013 for calculation of RR_aspect_. Positive values of RR_aspect_ indicate higher % cover values on S‐facing slopes, while negative values indicate higher % cover values on N‐facing slopes. Positive values of RR_drought_ indicate an increase in cover during the drought, while negative values indicate a decrease in cover. Results from a linear regression testing whether RR_drought_ is related to RR_aspect_ are provided for perennials. For annuals, we used an ANCOVA to determine the influence of RR_aspect_ and provenance on RR_drought_. Native and nonnative species exhibited similar relationships between RR_s_ (Provenance *F*_1,13_ = 0.02, *p *=* *.899, RR_aspect_ *F*_1,13_ = 5.67, *p *= .036)

There was a significant relationship between RR_drought_ and RR_aspect_ (Figure 3). Perennials that had greater cover on S‐facing than on N‐facing slopes also had a more positive response to the drought. The opposite pattern was observed for annuals and biennials, such that species that had greater cover on S‐facing slopes had a negative response to the drought. Both native and nonnative annuals and biennials exhibited similar relationships between RR_drought_ and RR_aspect_ (Figure 3).

## 4 Discussion

In this study, we evaluated the ability of spatial niches to predict patterns of vegetation response to a protracted drought. The idea that preferences in space can predict dynamics in time is currently utilized in many distributional models used to predict vegetation in future climate scenarios (Dormann, 2007; Elith & Leathwick, 2009). In our experiment, the direction of the relationship between drought response (RR_drought_) and aspect response (RR_aspect_) varied depending on the life cycle of the plant species. The fact that perennial and annual species showed opposite results may help to contextualize physiological, whole‐plant, and life history strategies for coping with water limitation. Water limitation is a universal factor in plant biology, where the photosynthetic compromise provides the basis for descriptions of the adaptive evolution of many plant processes, organs, and attributes (Cowan, 1982). The strategies for coping with drought expressed by perennial species in our study seem to be similar in space and time, while the strategies of shorter‐lived species seem to focus on escaping times of drought and taking advantage of brief, mesic periods in unoccupied spaces.

We expected species that preferred xeric slope aspects to have greater survivorship through the drought, because these species have developed strategies underlying this niche—tolerating, enduring, avoiding, or escaping dry conditions (Ackerly et al., 2002; Bochet, Garcia‐Fayos, & Poesen, 2009; Van de Water, Leavitt, & Betancourt, 2002). This hypothesis was supported only by the perennial species in our study. For example, *Eriogonum fasciculatum*, a drought‐deciduous shrub whose range extends from Central California south into Northwestern Mexico along the coast, and east into the Mojave Desert and Great Basin, had greater cover on S‐facing slopes, and was also able to continue to increase in cover during the drought. This pattern is in contrast to *Stipa pulchra*, a perennial bunchgrass with a range extending more into northern California and limited to more mesic coastal portions of Southern California, which had greater cover on N‐facing slopes and decreased in cover during the drought. Both of these patterns are consistent with the idea that spatial niche provides insight into performance associated with temporal variation in resource abundance. Interestingly, perennial species from this experiment exhibited an extremely tight, linear relationship between temporal and spatial patterns across the represented diversity in growth form (perennial grass vs. woody shrub), degrees of deciduousness, diversity of leaf forms (microphyll vs. macrophyll), and nitrogen‐fixing relationships (legume vs. nonlegume).

In contrast to the long‐lived species in our study, the annual and biennial species exhibited the opposite pattern, in which those with higher cover on S‐facing slopes decreased in cover during the drought to a greater extent than those with higher cover on N‐facing slopes. Along with this response, annual cover generally decreased through the drought, while perennial cover generally increased. The annuals that preferred S‐facing slopes primarily exhibited a drought‐escape strategy, because they remained dormant in the seed bank during dry years (Venable & Lawlor, 1980). The annual or biennial species that preferred more mesic slope aspects and managed to achieve greater cover than other annuals during the drought, such as *Phacelia cicutaria,* may have maintained higher cover because there was enough moisture on N‐facing slopes for germination and growth to occur every year. In contrast, the annual and biennial species that preferred S‐facing slopes, such as *Salvia columbariae*, had extremely low cover during the drought years. In contrast to the patterns seen in perennials, there was much more variance associated with the temporal and spatial responses associated with this life‐form, perhaps suggesting that shorter‐lived species encompass a greater diversity of functional strategies associated with water deficit compared to perennials.

Are the patterns seen in this study of a newly establishing community seen in mature communities with a more complex history of priority effects, diverse demographics, and legacies? The patterns we observed have been found in studies conducted in naturally established California grassland and coastal sage scrub systems, suggesting that the same dynamics operate in both restored and natural systems. Shrub species, such as *Eriogonum fasciculatum*, that exhibited a preference for one slope aspect or another in the West Loma Restoration Experiment, exhibited similar preferences in a naturally established coastal sage scrub system (Desimone & Burk, 1992). Additionally, the severe impact of drought on the perennial bunchgrass, *Stipa pulchra*, has been documented in natural and experimental settings (Hamilton, Holzapfel, & Mahall, 1999; Larios, Aicher, & Suding, 2013; Seabloom, 2007) along with the impact of drought on our suite of native forbs (Harrison, Gornish, & Copeland, 2015). The relative importance of abiotic and biotic differences in slope aspect likely changed through time in our restoration site as perennials grew and occupied more space. Such changes in the role of biotic interactions may be similar to postfire successional conditions in natural communities, in which seedlings compete for open spaces until shrubs become larger (Pacala & Tilman, 1994; Tyler, 1996). Switches in the direction of biotic interactions (along a scale between competitive and facilitative) likely also occurred as the drought progressed, because such interactions are known to vary depending on resource availability (Maestre, Callaway, Valladares, & Lortie, 2009; Padilla & Pugnaire, 2006).

Existing knowledge of the species in our study can be helpful for interpreting our results. For example, *E. fasciculatum* has drought‐tolerant traits such as deep roots, small leaves, and high WUE, along with drought‐enduring traits such as summer‐deciduous leaves (DeSimone & Zedler, 2001). In contrast, *S. pulchra* has more shallow roots than the perennial shrubs and is known to rely on water availability in the top 15–30 cm of soil (Hull & Muller, 1977). Although *S. pulchra* also exhibits leaf senescence during dry summers, it senesces later than annual grasses and has been identified as a species that requires more mesic habitats (Holmes & Rice, 1996). Seed dormancy in annuals is one trait that enables species to avoid dry years, yet dormancy may be unsuccessful for surviving prolonged drought (Harrison et al., 2015). Although non‐native species sometimes exhibit different responses than natives in the communities they invade (Funk & Vitousek, 2007; Kimball, Gremer, et al., 2014), the non‐native species in our system (all annuals) responded to drought and slope aspect in a similar manner to the native annual and biennial species. For example, non‐native *Erodium cicutarium* had higher cover on S‐facing than N‐facing slopes in the first 3 years of our study. However, by 2015, cover of this species was so low on S‐facing slopes that it actually had higher cover on N‐facing slopes, as exhibited by the significant year‐by‐aspect interaction.

On top of the cost efficiencies for research, one practical result of collecting data in a restoration project is that it provides critical information that practitioners may use in future restoration efforts. At our study site and in other semi‐arid temperate regions, prairie and shrubland coexist in a mosaic, with vegetation type‐conversions occurring between the two habitats (Archer, Schimel, & Holland, 1995; Kimball, Goulden, Suding, & Parker, 2014; Sankey, Ravi, Wallace, Webb, & Huxman, 2012). Most of the prairie species in our restoration project preferred N‐facing slopes, while the shrubland (coastal sage scrub) species were more mixed in their preference for N‐ and S‐facing slopes. Given that practitioners are often confronted with more land to restore than funds to support restoration, we recommend that restoration practitioners select N‐facing slopes for the restoration of California prairie communities. This would result in a greater response of plant cover and communities more resilient to drought per unit dollar invested in such projects. In addition, we recommend a species‐specific approach, and analyzing data from restoration projects conducted in other areas will allow the development of lists of species or communities that may have greater establishment in dry years or on xeric slope aspects.

The application of spatial niche preferences to predict abundance in time has been applied with some controversy in niche modeling, with the goal of using existing range information to predict response to future climate change (Dormann, 2007; Elith & Leathwick, 2009). With niche modeling, researchers have cautioned that biotic interactions, evolution in response to novel stressors, and dispersal limitations may influence predictability (Dormann, 2007). By seeding species simultaneously across possible spatial niches, we overcame potential barriers to seed dispersal. Additionally, differences in biological interactions were minimized by weeding at standardized rates across sites. In our attempt to predict response to drought through spatial niche preference, we found that the life cycle of the plant determined direction of response, indicating the importance of understanding how different growth strategies influence ability to tolerate specific spatial environmental conditions.

Trait‐based studies have also attempted to predict how species may respond to future conditions, with the idea that certain trait combinations may allow species to increase or decrease in response to warming or drought (Gornish & Prather, 2014; McGill, Enquist, Weiher, & Westoby, 2006; Violle, Reich, Pacala, Enquist, & Kattge, 2014). While traits (such as those that allow for drought tolerance or escape) may aid in predicting response, our results recommend considerations of both seed and canopy dormancy in understanding such predictions. Other studies of the influence of drought have found that response varied with slope aspect, suggesting the importance of determining how different slope aspects may exhibit different thresholds for vegetation change (Bennie, Hill, Baxter, & Huntley, 2006; Elliott & Cowell, 2015). Our results, which included significant year‐by‐aspect interactions for several species, support this idea that slope aspect influences the response of vegetation to drought. What is evident from these data is that categorizing any one of these species into the historically used drought strategies (e.g., avoidance, escape, tolerance, endurance) could be problematic—as illustrated by interplay of spatial and temporal responses to water deficit. This is due to the many combinations of physiological, organismal, life history, or population strategies that are employed by a given species to deal with the multiple temporal and spatial scales over which water deficit may be expressed. Progress in efforts to relate functional traits to population and community dynamics in response to real ecosystem drivers, such as drought, will have to develop means to integrate these complex responses across the hierarchy of life.

In conclusion, the growing number of large restoration projects across landscapes, involving similar species and systems, provides opportunities to investigate spatial niche preferences. Such niche preferences may be combined with developing theory underlying the relationship of species’ traits and strategies for persisting and functioning through stress to make more robust predictions regarding plant and ecosystem response to future stressors. Specifically, preference for slope aspect may be useful in predicting response to drought, with potentially different responses depending on life cycle stages employed in enduring, avoiding, escaping or tolerating water stress. For perennial species, the ability to tolerate xeric spatial conditions corresponded to an ability to tolerate drought, while for annuals species, a preference for xeric slope aspects resulted in less germination during drought. The current challenge is to build careful considerations of life cycle and drought response strategies into models that use spatial niche preferences to predict response to climate change.

## Conflict of Interest

None declared.

## Author Contributions

SK, ML, and TH conceived of and designed the study. KB collected the data and conducted initial data analyses. SK analyzed the data. SK wrote the first draft of the manuscript, and all authors contributed substantially to revisions.
