# Supplementary material for: Predicting drought tolerance from slope aspect preference in restored plant communities
Source: Ecol Evol. 2017 Mar 30;7(9):3123–31. doi: 10.1002/ece3.2881 (PMC5415533; doi:10.1002/ece3.2881)
Supplement: Supplementary file 1 [file ECE3-7-3123-s001.docx]

APPENDIX 1

**
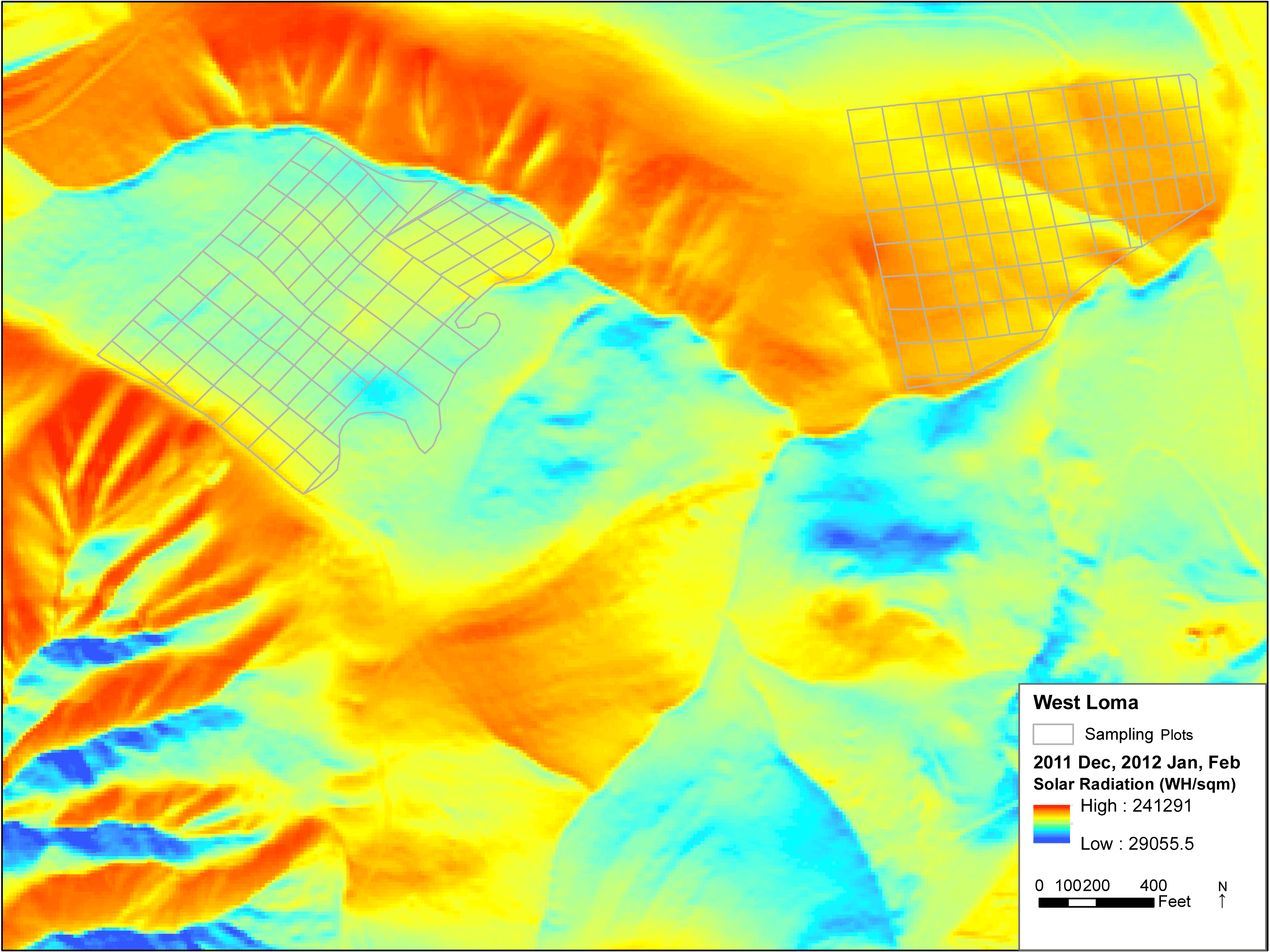
**

Map of the West Loma Ecological Restoration Experiment study area, showing the amount of solar radiation received on N- and S-facing slopes during the winter months. Sampling areas are outlined in gray. The same seed mixes were applied to both slope aspects.

APPENDIX 2

RR_aspect_ values calculated in each year. We used 2013 values in our analyses comparing aspect response to drought response because this choice allowed time for environmental differences to influence abundance, yet avoided the later years that likely reflected severe drought conditions.

| **Species** | **Code** | **Native** | **Lifecycle** | **2012** | **2013** | **2014** | **2015** |
| --- | --- | --- | --- | --- | --- | --- | --- |
| *Artemisia californica* | ARTCAL | Yes | P | 0.335 | -0.071 | -0.300 | -0.224 |
| *Elymus condensatus* | ELECON | Yes | P | -1.312 | -0.494 | -8.509 | 0.435 |
| *Encelia californica* | ENCCAL | Yes | P | -0.472 | -0.405 | -0.541 | -0.615 |
| *Eriogonum fasciculatum* | ERIFAS | Yes | P | 0.210 | 0.390 | 0.255 | 0.243 |
| *Isocoma menziesii* | ISOMEN | Yes | P | -0.486 | 0.682 | 0.807 | 0.138 |
| *Salvia apiana* | SALAPI | Yes | P | -0.377 | 0.077 | -0.144 | -0.391 |
| *Stipa pulchra* | STIPUL | Yes | P | -0.849 | -0.927 | -0.579 | -1.149 |
| *Deinandra fasciculata* | DEIFAS | Yes | A | 0.041 | -0.602 | 3.440 | -0.888 |
| *Eschscholzia californica* | ESCA | Yes | A | -0.236 | 8.498 | 1.719 | -0.371 |
| *Grindelia camporum* | GRICAM | Yes | A | -0.206 | 0.044 | -0.082 | -1.063 |
| *Malacothrix saxatilis* | MASA | Yes | A | 0.064 | 0.256 | 2.128 | 1.297 |
| *Phacelia cicutaria* | PHCI | Yes | A | -1.035 | -1.398 | -0.380 | -2.079 |
| *Plantago erecta* | PLAERE | Yes | A | -0.419 | -2.293 | 2.636 | -1.988 |
| *Salsola tragus* | SACO | Yes | A | 2.409 | 1.049 | 1.513 | 0.978 |
| *Brachipodium distachyon* | BRADIS | No | A | 0.592 | -0.614 | 0.226 | -1.527 |
| *Brassica nigra* | BRANIG | No | A | -0.345 | 1.066 | 0.582 | -0.779 |
| *Bromus madritensis* | BROMAD | No | A | 7.637 | -0.063 | 1.324 | -0.335 |
| *Erodium cicutarium* | EROCIC | No | A | 1.232 | 1.168 | 2.171 | -1.105 |
| *Melilotus indicus* | MELIND | No | A | -2.139 | -1.045 | -0.267 | -0.737 |
| *Salvia columbariae* | SALTRA | No | A | 1.108 | 1.895 | 3.349 | 1.145 |
| *Sonchus oleraceus* | SONOLE | No | A | -0.209 | 0.699 | 1.612 | 0.071 |

APPENDIX 3

Results of repeated measures, mixed model ANOVAs testing the influence of slope aspect, year, and the aspect-by-year interaction on % cover of each species.

| **Native Species** | **Effect** | **Num DF** | **Den DF** | **F** | **P** |
| --- | --- | --- | --- | --- | --- |
| *Acmispon strigosus* | Year | 3 | 864 | 115.03 | <.0001 |
|  | Aspect | 1 | 864 | 8.84 | 0.003 |
|  | Year*Aspect | 3 | 864 | 10.63 | <.0001 |
| *Artemisia californica* | Year | 3 | 611 | 22.71 | <.0001 |
|  | Aspect | 1 | 611 | 0.13 | 0.7158 |
|  | Year*Aspect | 3 | 611 | 3.28 | 0.0207 |
| *Deinandra fasciculata* | Year | 3 | 612 | 18.69 | <.0001 |
|  | Aspect | 1 | 612 | 5.9 | 0.0155 |
|  | Year*Aspect | 3 | 612 | 0.84 | 0.4741 |
| *Elymus condensatus* | Year | 3 | 612 | 14.44 | <.0001 |
|  | Aspect | 1 | 612 | 1.7 | 0.1927 |
|  | Year*Aspect | 3 | 612 | 1.56 | 0.197 |
| *Encelia californica* | Year | 3 | 611 | 14.26 | <.0001 |
|  | Aspect | 1 | 611 | 3.93 | 0.048 |
|  | Year*Aspect | 3 | 611 | 3.28 | 0.0205 |
| *Eriogonum fasciculatum* | Year | 3 | 611 | 159.17 | <.0001 |
|  | Aspect | 1 | 611 | 8.2 | 0.0043 |
|  | Year*Aspect | 3 | 611 | 2.88 | 0.0353 |
| *Eschscholzia californica* | Year | 3 | 612 | 201.81 | <.0001 |
|  | Aspect | 1 | 612 | 0.07 | 0.7943 |
|  | Year*Aspect | 3 | 612 | 2.72 | 0.0439 |
| *Grindelia camporum* | Year | 3 | 864 | 22.2 | <.0001 |
|  | Aspect | 1 | 864 | 2.07 | 0.1505 |
|  | Year*Aspect | 3 | 864 | 0.53 | 0.6641 |
| *Isocoma menziesii* | Year | 3 | 864 | 10.42 | <.0001 |
|  | Aspect | 1 | 864 | 0.04 | 0.8448 |
|  | Year*Aspect | 3 | 864 | 0.44 | 0.7217 |
| *Malacothrix saxatilis* | Year | 3 | 612 | 8.53 | <.0001 |
|  | Aspect | 1 | 612 | 2.11 | 0.1465 |
|  | Year*Aspect | 3 | 612 | 1.79 | 0.1484 |
| *Phacelia cicutaria* | Year | 3 | 612 | 97.04 | <.0001 |
|  | Aspect | 1 | 612 | 18.88 | <.0001 |
|  | Year*Aspect | 3 | 612 | 36.71 | <.0001 |
| *Plantago erecta* | Year | 3 | 864 | 12.59 | <.0001 |
|  | Aspect | 1 | 864 | 11.53 | 0.0007 |
|  | Year*Aspect | 3 | 864 | 12.55 | <.0001 |
| *Salvia apiana* | Year | 3 | 611 | 34.12 | <.0001 |
|  | Aspect | 1 | 611 | 3.28 | 0.0706 |
|  | Year*Aspect | 3 | 611 | 2.36 | 0.0708 |
| *Salvia columbariae* | Year | 3 | 612 | 39.68 | <.0001 |
|  | Aspect | 1 | 612 | 19.21 | <.0001 |
|  | Year*Aspect | 3 | 612 | 4.35 | 0.0048 |
| *Stipa pulchra* | Year | 3 | 864 | 86.94 | <.0001 |
|  | Aspect | 1 | 864 | 27.57 | <.0001 |
|  | Year*Aspect | 3 | 864 | 3.11 | 0.0258 |
| **Non-native Species** | **Effect** | **Num DF** | **Den DF** | **F** | **P** |
| *Brassica nigra* | Year | 3 | 4499 | 143.6 | <.0001 |
|  | Aspect | 1 | 4499 | 0.32 | 0.5711 |
|  | Year*Aspect | 3 | 4499 | 16.84 | <.0001 |
| *Brachipodium distachyon* | Year | 3 | 4499 | 114.41 | <.0001 |
|  | Aspect | 1 | 4499 | 0.61 | 0.4346 |
|  | Year*Aspect | 3 | 4499 | 24.92 | <.0001 |
| *Bromus madritensis* | Year | 3 | 4499 | 9.78 | <.0001 |
|  | Aspect | 1 | 4499 | 0.24 | 0.6223 |
|  | Year*Aspect | 3 | 4499 | 0.51 | 0.6774 |
| *Erodium cicutarium* | Year | 3 | 4499 | 42.21 | <.0001 |
|  | Aspect | 1 | 4499 | 7.23 | 0.0072 |
|  | Year*Aspect | 3 | 4499 | 16.82 | <.0001 |
| *Melilotus indicus* | Year | 3 | 4499 | 59.15 | <.0001 |
|  | Aspect | 1 | 4499 | 19.46 | <.0001 |
|  | Year*Aspect | 3 | 4499 | 14.79 | <.0001 |
| *Salsola tragus* | Year | 3 | 4499 | 69.7 | <.0001 |
|  | Aspect | 1 | 4499 | 34.1 | <.0001 |
|  | Year*Aspect | 3 | 4499 | 26.39 | <.0001 |
| *Sonchus oleraceus* | Year | 3 | 4499 | 113.78 | <.0001 |
|  | Aspect | 1 | 4499 | 4.76 | 0.0292 |
|  | Year*Aspect | 3 | 4499 | 12.08 | <.0001 |

APPENDIX 4


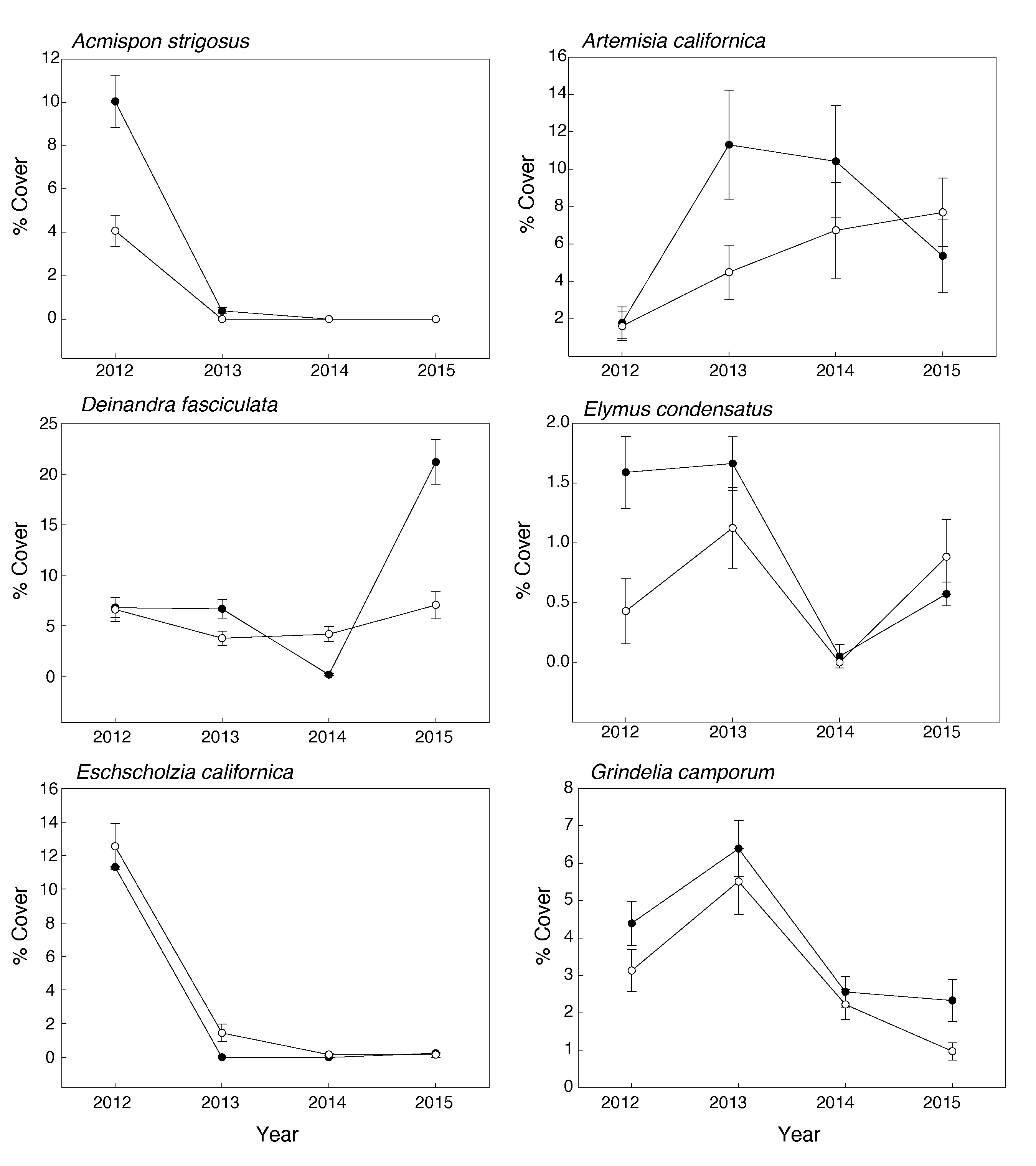


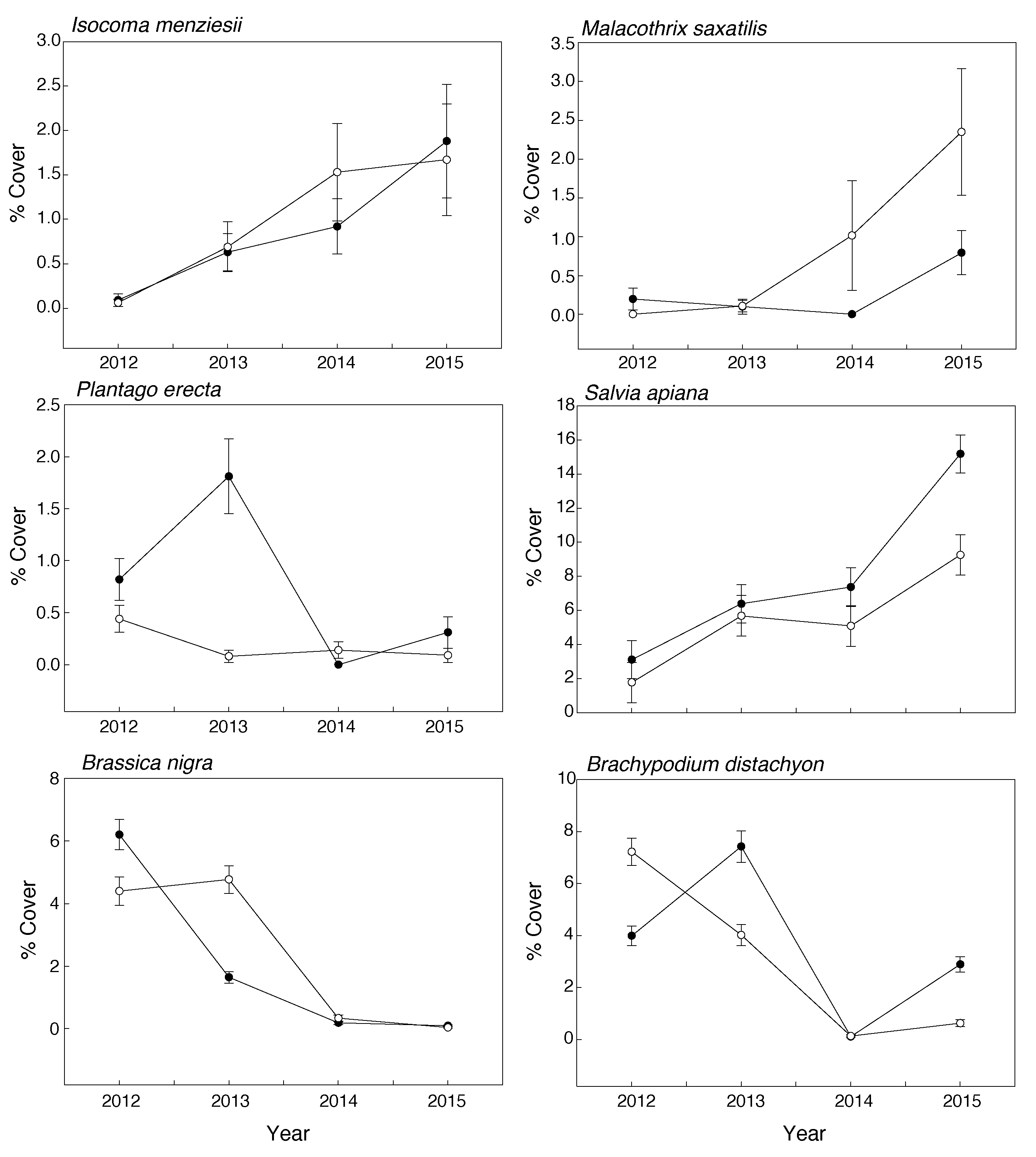


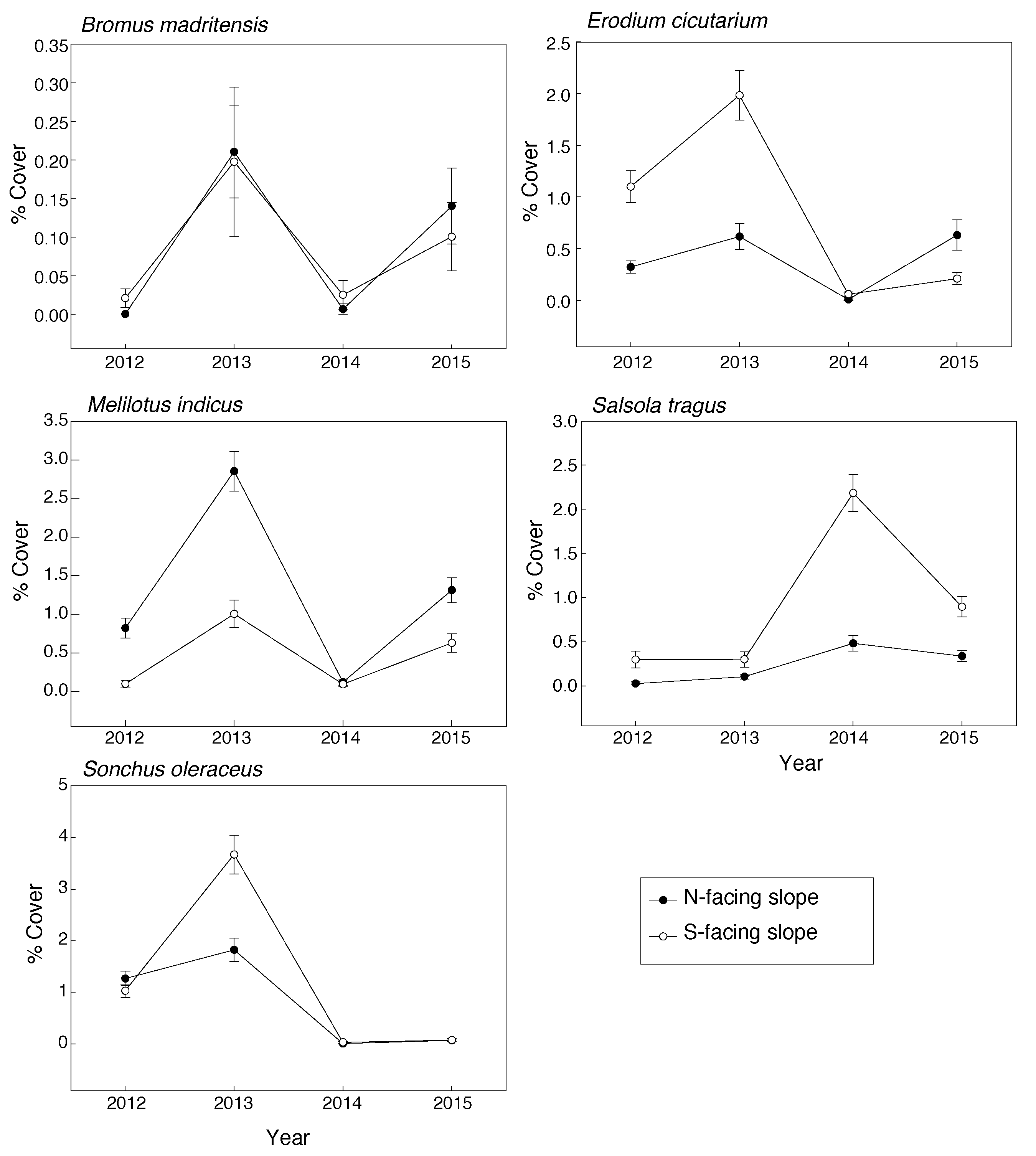


The % cover of species through time on N-facing and S-facing slopes. Graphs of abundant species are provided in Fig. 2 of the manuscript. Results from ANOVAs of all species are provided in APPENDIX 3.
